# Analyzing the Interaction between *Tetrahymena pyriformis* and Bacteria under Different Physicochemical Conditions When Infecting Guppy Using the eDNA Method

**DOI:** 10.3390/ani14152194

**Published:** 2024-07-27

**Authors:** Jialu Wang, Xiaosong Wang, Lihui Liu, Xiang Wang, Jiarui Wang, Yue Zheng, Li Wang, Xuming Pan

**Affiliations:** Laboratory of Protozoology, Harbin Normal University, Harbin 150025, China; hsdzqy@stu.hrbnu.edu.cn (J.W.); hsdybj@stu.hrbnu.edu.cn (X.W.); hsdlmh6778@stu.hrbnu.edu.cn (L.L.); hsdgjt@stu.hrbnu.edu.cn (X.W.); hsdykx@stu.hrbnu.edu.cn (J.W.); hsdtj@stu.hrbnu.edu.cn (Y.Z.); hsd6228@hrbnu.edu.cn (L.W.)

**Keywords:** ciliated protozoan, aquaculture, bacterial microbiome, environmental DNA, fish disease

## Abstract

**Simple Summary:**

In this study, environmental DNA technology, 16S rRNA gene sequencing, and quantitative reverse transcription polymerase chain reaction (qRT-PCR) were employed to investigate the relationships between *Tetrahymena pyriformis*, bacteria, and guppies under varying temperature and pH conditions. The abundance of *T. pyriformis* in water, the relative abundance of bacterial species, and histopathological observations were studied. Results of this study showed that several bacteria were related to *T. pyriformis*, and environmental factors affected the interaction between bacteria and *T. pyriformis*.

**Abstract:**

In the aquaculture system of ornamental fish, the interaction between bacterial microbiota and ciliate protozoa can prevent or promote disease outbreaks, and different physicochemical conditions will affect the relationships between them. We investigated the interaction between bacterial microbiota and the parasite *Tetrahymena pyriformis* when infecting *Poecilia reticulata* (guppy) under different physicochemical conditions. The abundance of *T. pyriformis* in water, the relative abundance of bacterial species, and histopathological observation were studied or monitored using environmental DNA (eDNA) extraction technology, the qPCR method, and 16s rRNA sequencing, respectively. The morphological identification and phylogenetic analysis of *T. pyriformis* were carried out. The infected guppy tissue was also stained by the hematoxylin and eosin methods. The results showed: (1) the bacterial communities of water samples were mainly composed of species assigned to Proteobacteria and Bacteroidetes, and *Tabrizicola* and Puniceicoccaceae were positively correlated with fish mortality, *T. pyriformis* abundance, and temperature. (2) *Arcicella* and *Methyloversatilis* universalis with different correlations between ciliates appeared in different treatment groups, the result of which proved that environmental factors affected the interaction between bacteria and *T. pyriformis*. (3) Lower temperatures and a higher pH were more beneficial for preventing disease outbreaks.

## 1. Introduction

Ornamental fish aquaculture is one of the most valuable aquaculture industries [1,2]. Some diseases, occasionally accompanied by mass mortalities, have occurred in guppy markets; research on guppy diseases is scant [3,4]. The collective name for diseases of vertebrate and invertebrate hosts caused by *Tetrahymena* is tetrahymenosis [5]. Tetrahymenosis is a common disease in guppy [6,7,8]. In previous studies, it was found that *T. pyriformis* could easily infect guppies, especially those injured in transportation, resulting in casualties, which caused huge losses to aquarium owners [9,10,11]. *Tetrahymena pyriformis* is the killer of guppies [12,13,14].

Environmental factors affect the species composition as well as the abundance of microorganisms in the water and increase the risk of disease in cultured ornamental fish [15,16]. Culture water temperature and pH in the in-house ornamental market are commonly variables in the culture process [17]. Recent studies have revealed the close relationship between bacterial microflora and parasitic ciliates in the aquatic environment [16,18]. However, the interaction between *T. pyriformis* and bacterial microorganisms under different physical factors has not been studied. The interaction between the bacterial microbiome and parasitic *Tetrahymena* species in aquaculture systems can prevent or promote the outbreak of disease. At the same time, physical and chemical factors also affect the occurrence of the disease [19,20].

Environmental DNA extraction technology can be described as the method by which DNA is extracted from environmental samples (water, soil, sediment, air, mixtures, etc.). Using eDNA extraction technology to detect the abundance of parasitic ciliates and microorganisms in water, we can easily find out the factors affecting the death of fish [21,22,23,24,25,26]. Environmental DNA extraction technology can be used to monitor the amounts and species of fish, parasites, and related bacterial microflora in aquaculture water.

In our work, we utilized eDNA extraction technology and qPCR to investigate the relationships among guppies, *T. pyriformis*, and related bacterial microflora under different temperature and pH conditions. Additionally, we employed hematoxylin-eosin (HE) staining and cultivable bacteria on a plate to confirm the infection of the ciliate and related bacterial microflora. Through bioinformatics analysis, we investigated the interaction between bacteria and *T. pyriformis* at different temperatures and pH levels with the aim of enhancing the ornamental fish’s survival rate during transportation or cultivation.

## 2. Materials and Methods

**Figure 1:**
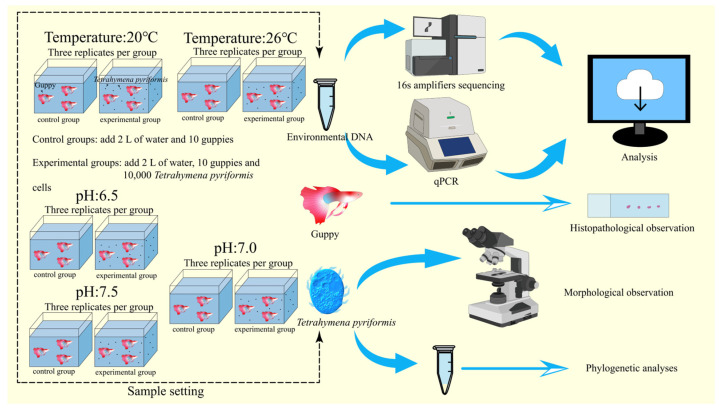
Experimental design illustration.

The Complete Experimental Design is Illustrated in Figure 1.

### 2.1. Sample Setting

The heating device (Jiale LN27-1000, Jiale Scientific Instruments Co., Ltd., Xiamen, China) was used to set up two groups of simulated aquatic environments at 20 °C and 26 °C in the laboratory [27]. In order to eliminate the interference of other parasites, we boiled tap water and used it in the coming experiments after it had cooled. The experimental groups and the control groups were set up, and each group had 2 L of water and 10 guppies. Guppies of the same species were sourced from a single aquarium and were not provided with feed following the commencement of the treatment. The average weight of the guppy individuals was 10 g, and the average length was 4 cm. Ten thousand *T. pyriformis* cells (20 mL × 500 ind./mL) were added to the water of the experimental group while the control group had no *T. pyriformis* cells to ensure that the guppies in the control group were not infected by *T. pyriformis*. An oxygen pump was added to each tank, maintaining an average dissolved oxygen level of 7.30 mg/L. Both the control groups and the experimental groups were repeated three times.

In the laboratory, simulated aquatic environments were established with three pH levels: 6.5, 7.0, and 7.5 [28,29]. PH was adjusted by HCl and NaOH, and it was measured by a pH instrument (LeiCi PHS-25, Shanghai Rex Instrument Factory, Shanghai, China). In each treatment group, 2 L of water and 10 guppies were added. The control group lacked *T. pyriformis*, while the experimental group received an inoculation of 10,000 cells of *T. pyriformis*. An oxygen pump was added to each tank, maintaining an average dissolved oxygen level of 7.30 mg/L. Both the control groups and the experimental groups were repeated three times (Figure 1).

The animal experiments were conducted following the Guide for the Care and Use of Laboratory Animals and the Harbin Normal University Approval for Research Involving Animals (No. HNUARIA2024008). To simulate the actual conditions of guppies during transportation, we scraped the skin of each fish in the control and experimental groups with a disinfected blade, creating average incisions of 1 cm in length and 0.5 mm in depth. In accordance with the Guide for the Care and Use of Laboratory Animals, the fish were anesthetized with tricaine methane-sulfonate (MS-222) prior to incision.

### 2.2. Ciliate Isolation, Cultivation, Morphological Identification, DNA Extraction, and Sequencing

When viewing under a stereomicroscope, we used a clean micropipette to separate multiple *T. pyriformis* cells from the gills and mucus of infected guppies. Monoclonal *T. pyriformis* was established in sterile RM-9 culture medium in a Petri dish (20 mL) and maintained at 25 °C. Cells were counted using a hemocytometer(Hausser, Beijing, China). Field observation was performed under a microscope with phase contrast illumination (Zeiss Axio imager A2, Carl Zeiss AG, Oberkochen, Germany). Silver carbonate staining methods were used to reveal the infraciliature of the nuclear apparatus [30]. Counts and measurements of silver-stained specimens were performed at magnifications of 100× to 1000×. Classification and terminology are according to Lynn [31].

A single cell of *T. pyriformis* was washed individually with distilled water. Genomic DNA was extracted from five pooled cells using a DNeasy Blood & Tissue Kit (Qiagen, Hilden, Germany), following the manufacturer’s instructions. The *cox1* gene was amplified with the primers F298dT (50-TGTAAA ACG ACG GCC AGT GCN CAY GGTYTA ATN ATGGT-30) and R1184dT (50-CAG GAA ACA GCT ATG ACT ADA CYT CAG GGT GAC CRA AAA ATC A-30). Bidirectional sequencing was performed by the Shanghai Sunny Biotechnology Company (Shanghai, China). The *Cox1* gene sequences were downloaded from NCBI and aligned with those of the newly sequenced *T. pyriformis*. After alignment, both primer sequences were trimmed using Bioedit 7.0.1 [32]. The numbers of unmatched sites were counted one by one, then both ends were trimmed and pairwise comparisons were made.

### 2.3. Phylogenetic Analyses

Bayesian inference (BI) analysis was carried out with MrBayes (v3.2.6) on XSEDE v3.2.6 on CIPRES Science Gateway (http://www.phylo.org/, accessed on 20 July 2024) using the GTR + I + G evolutionary model as the best-fit model selected by MrModeltest v.2 according to the Akaike Information Criterion (AIC) [33,34,35]. A Maximum Likelihood (ML) tree was constructed using RAxML-HPC2 v. 8.2.10 on the CIPRES Science Gateway with the optimal model GTR + I + G evolutionary model as the best model according to the AIC criterion selected by the program Modeltest v.3.4 [35,36]. Node support came from 1000 bootstrap replicates. TreeView v.1.6.6 and MEGA v5 were used to visualize tree topologies [37].

### 2.4. Environmental DNA Extraction and 16s Amplifiers Sequencing

Each sample was filtered with a 0.22 μm membrane made of polyether sulfone. Total DNA was extracted from the samples following the manufacturer’s instructions, utilizing the DNeasy Power Water Kit from QIAGEN, Germany. EDNA was extracted from the experimental groups and the control groups after more than half of the fish died.

The full-length region of 16s was amplified by extracting DNA from the sample. After comparing the concentration of PCR products using Gene Tools Analysis Software (Version 4.03.05.0, Syn Gene, Bengaluru, Karnataka, India). The required volume of each sample was calculated according to the equal mass principle, and each PCR product was mixed. The mixed PCR products were recovered by the Hi Pure Gel Pure DNA Mini Kit (Megen Biotech, Shanghai, China) gel recovery kit, and the target DNA fragments were eluted with TE buffer. The database was built according to the standard process of 16s Amplification SMR Tbell Library Preparation, and the amplified sublibrary was sequenced by the PacBio Sequel II platform.

### 2.5. qPCR Amplification

In order to measure the abundance of *T. pyriformis*, the *β-tubulin* gene of *T. pyriformis* in the eDNA of the control group and the experimental group was detected by qPCR. To avoid contamination, pipettes, straw heads, and gloves were sterilized with ultraviolet light for 30 min on a clean bench before use. QPCR was performed on a CFX96 Multicolor Real-Time PCR Detection System (Bio-Rad Laboratories, Hercules, CA, USA) using the Ultra SYBR Mixture (Beijing Com Win Biotech, Beijing, China) with the 18S rRNA gene as the reference gene. In order to ensure the activity of the sample, the sample addition process was protected from light and carried out on ice. The real-time PCR reaction conditions were as follows: 95 °C, reaction time 30 s, 95 °C 10 s, 56 °C 30 s, cycle 40 times, and the dissolution curve were determined by increasing the rate of 0.5 °C for 5 s in the range of 60 °C~90 °C. The melting curve was analyzed at the end of the amplification cycle to determine the specificity of the PCR products. The gene expression levels were calculated by the 2^−ΔΔCT^ method [38].

### 2.6. Histopathological Observation

Tissue sections of muscle, gill, heart, liver, spleen, eye, stomach, intestine, and other organs or diseases of infected and healthy guppies were carried out to investigate the tissue damage and existence of *T. pyriformis*. The tissues of the extracted guppies in Bouin’s solution were fixed for 24–48 h and stored in 70% alcohol. The treated tissue was gradually dehydrated in a 70–100% alcohol solution, then the sample was soaked in a 1:1 solution of xylene and ethanol, a 1:1 solution of xylene and paraffin wax for 1 h, and finally ran into paraffin wax and wrapped in paraffin wax. The embedded tissues were cut into thin slices with a thickness of 5–7 μm and put into the oven at 60 °C for 30 min. The preheated tissue sections were stained with hematoxylin-eosin. Finally, the stained tissue sections were examined under a light microscope.

### 2.7. Microbial Composition in Cultural Water

The microflora of water samples was detected. All the relevant articles were sterilized in a sterilizing pot. The water samples of 100 μL were absorbed from the clean bench, coated on the plate with LB solid medium, and cultured at 37 °C without light for 24 h. The bacteria with different morphologies were selected and purified on LB solid medium. After purification three times, the single colonies were crossed into LB solid-slope culture medium. The purified strains were picked into the EP tube of 2 mL with LB liquid medium and shaken on a shaker for 24 h. Under aseptic operation conditions, 500 μL bacteria solution and 500 μL 50% glycerol solution were added to the sterilized 2 mL cryopreservation tube, gently mixed, stored at −80 °C, and the strain was retained.

### 2.8. Statistical Analysis and Bioinformatics

Data from a total of 30 samples, including three replicates each of the control and experimental groups under treatments of temperatures of 20 °C and 26 °C and pH levels of 6.5, 7.0, and 7.5, were analyzed. The original 16S sequence was trimmed using fast preprocessing (fastp), resulting in 69,624 sequences [7]. Sequences were assigned to samples based on labels and barcodes. USEARCH v9.2 was used to remove barcode sequences and potential chimerism [39]. Sequences with 97% similarity were aggregated into operational taxonomic units (OTUs) according to the standard set in USEARCH v9.2.

The dilution curve of the reads from all samples, treated at temperatures of 20 °C and 26 °C and at pH levels of 6.5, 7.0, and 7.5, was calculated as a test of whether the sample size is sufficient to ensure there is adequate sample depth for follow-up analysis. A one-way ANOVA was conducted using R’s aov () function to assess differences among sample groups treated under varying temperature and pH conditions [40]. Upon detecting significant differences, a *t*-test was initially applied, followed by Duncan’s multiple range test for post hoc analysis, with the level of significance set at *p* < 0.05. The Alpha diversity of the bacteria matrix was visualized using the “ggplot2” package using R software (R version 4.3.0). The “networkD3” and “ggplot2” packages of R software are used to generate Sankey diagrams, box diagrams, and taxon summary bar charts. The correlation between *Tetrahymena pyriformis*, bacteria, and guppies under varying temperature and pH conditions was compared, and the correlation heat map was drawn using ChiPlot, available at https://www.chiplot.online/ (accessed on 20 July 2024).

## 3. Results

### 3.1. Histopathology of Guppies

**Figure 2:**
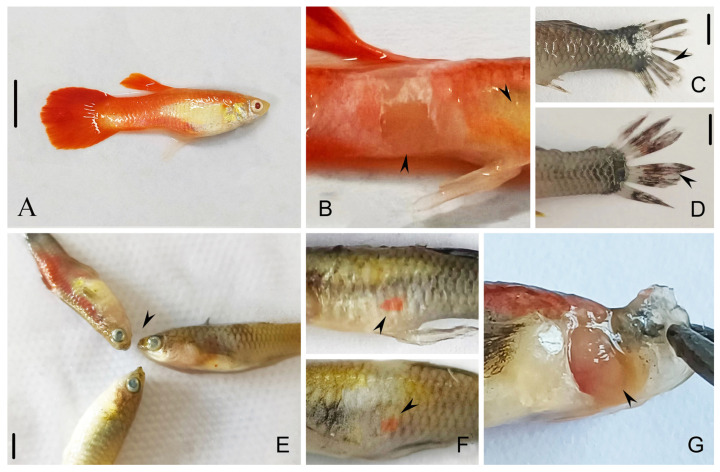
The pathological features of healthy guppy (**A**) and diseased guppy (**B**–**G**). (**A**) The holistic view of a healthy fish. (**B**) Body surface with skin falling off and festering (arrowheads). (**C**,**D**) The seriously damaged tail of the diseased fish (arrowheads). (**E**) Diseased fish with gray eyes (arrowhead). (**F**) Fish with erythema appearing on the surface (arrowheads). (**G**) Congested and swollen gill of the diseased fish (arrowhead). Scale bars = 1 cm (**A**), 5 mm (**C**,**D**), 4 mm (**E**).

**Figure 3:**
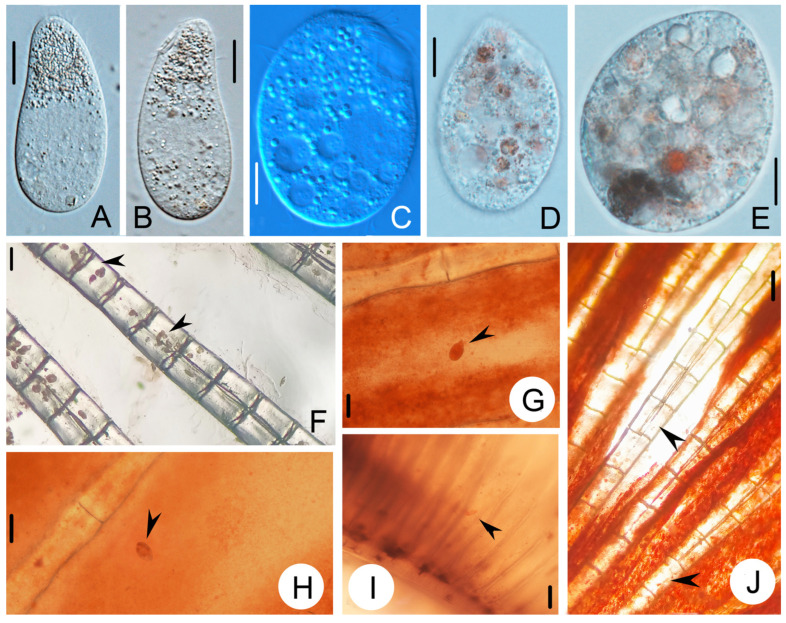
Photomicrographs of *Tetrahymena pyriformis* from life. (**A**,**B**) Photomicrograph showing *T. pyriformis* that has not yet infected fish. (**C**–**E**) Photomicrograph of *Tetrahymena pyriformis* found on the body surface of a diseased fish. (**F**–**I**) Fin of diseased guppy, showing the presence of *T*. *pyriformis*. (arrowheads). (**J**) *Tetrahymena pyriformis* is found in the gill of the fish (arrowhead). Scale bars = 10 μm (**A**,**B**,**D**,**E**), 20 μm (**C**), 80 μm (**F**–**J**).

*Tetrahymena pyriformis* infects guppy tissue (e.g., gills, liver, and muscle tissue), gains entry through skin injuries, and subsequently spreads throughout the entire fish body. Compared with the fish of uninfected *T. pyriformis* (Figure 2A), infected fish gradually lost their vitality, leading to an inability to maintain balance, ultimately culminating in death. Compared with healthy guppy (Figure 2A), the gills of affected fish exhibited congestion and ulceration (Figure 2G). Some diseased fish had gray eyeballs (Figure 2E), dark red spots in the subcutaneous tissue of the body (Figure 2F), and a lot of mucus (Figure 2B). The tails of some diseased fish were seriously damaged (Figure 2C,D). Under the light microscope, many *T. pyriformis* could be directly observed in the fresh specimens extracted from the gill and fin tissues (Figure 3F–J). In the fin, the ciliates moved moderately (Figure 3F,G,J). In vivo observation of these *T. pyriformis* revealed the presence of fish pigment particles in their food vesicles (Figure 3D,E).

**Figure 4:**
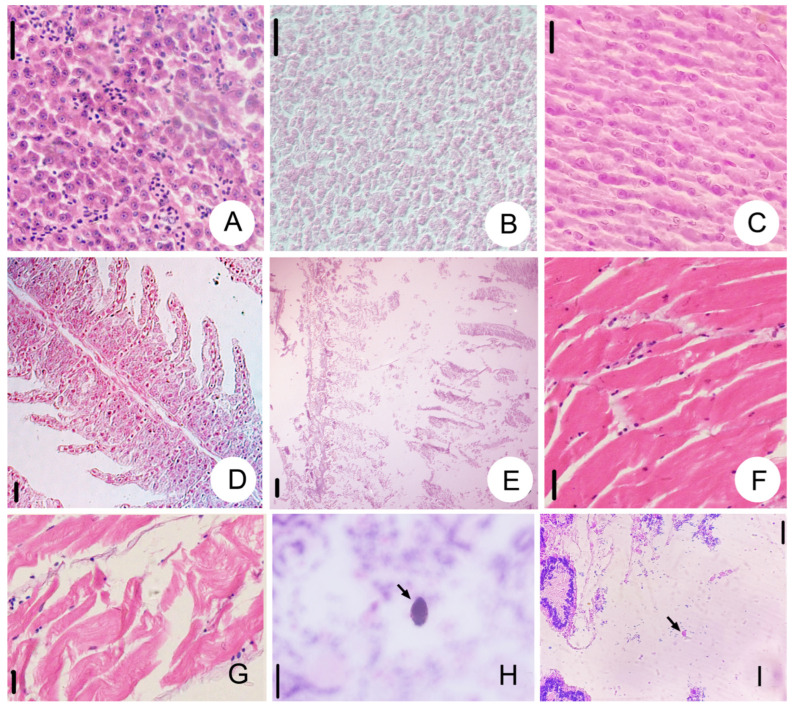
Histological sections of guppy liver (**A**–**C**), gill (**D**,**E**) and muscle tissue (**F**–**I**) stained by hematoxylin and Eosin. (**A**) The liver tissue of the control group fish (**B**,**C**) Vacuolization of liver tissue of fish from experiment groups. (**D**) The gill of the control group fish. (**E**) The gill tissue of diseased fish atrophied and ruptured. (**F**) The muscle tissues from the fish in the control group. (**G**) The muscle tissue of diseased fish atrophied and ruptured. (**H**,**I**) *Tetrahymena pyriformis* is found in muscle tissue (arrows). Scale bars = 200 μm (**A**–**E**), 80 μm (**F**–**I**).

The organs or tissues of guppies were histologically examined by hematoxylin-eosin staining. Compared with the liver cells of fish from control groups (Figure 4A), those of the diseased guppies were vacuolated and discontinuous (Figure 4B,C). Compared with the gills of fish from control groups (Figure 4D), the gills of the diseased fish were atrophied, the gill filament was broken, and the gill was seriously damaged (Figure 4E). In addition, the muscle tissues of diseased fish atrophied and ruptured (Figure 4G), while those from control groups were unchanged (Figure 4F). Cells of *Tetrahymena pyriformis* were found in the sections of muscle and intestinal tissue of diseased fish (Figure 4H,I).

When the extracted eDNA water samples were applied to LB medium, it was found that there were more colonies in the temperature treatment group at 26 °C. In the pH treatment group, the bacterial colonies at pH 6.5 and 7.5 were higher than 7.0. The colonies were purified and preserved for subsequent identification. After conducting an F-test on the bacterial abundance, we obtained a *p*-value of 0.001, which indicated a highly significant result.

### 3.2. Morphological Description of the Harbin Population of Tetrahymena pyriformis

Cells are about 50 µm × 20 µm in vivo in common; the body is elongated in outline; the buccal area is small and sub-apically located (Figure 3A,B). When infected guppies, *T. pyriformis* plumps out about 50 µm × 35 µm (Figure 3C–E). Cytoplasm is colorless to slightly grayish and packed with large amounts of round granules (1–3 µm in diameter), bacteria-filled food vacuoles, and irregularly shaped crystals (Figure 3C–E). The somatic cilia are sparsely arranged. No caudal cilium. One spherical macronucleus, approximately 15 μm across, is positioned at the body center (Figure 3D,E). The micronucleus was not observed. A single contractile vacuole is about 5 µm in diameter. Locomotion means swimming moderately fast, sometimes lying motionless along the substrate. Macrostomes are produced when bacterial food is depleted (Figure 3E). Twenty or 21 somatic kineties. There are two postoral kineties. There are three parallel membranelles, each with three rows of basal bodies.

### 3.3. Phylogenetic Analyses Based on Cox1 Gene Sequences

**Figure 5:**
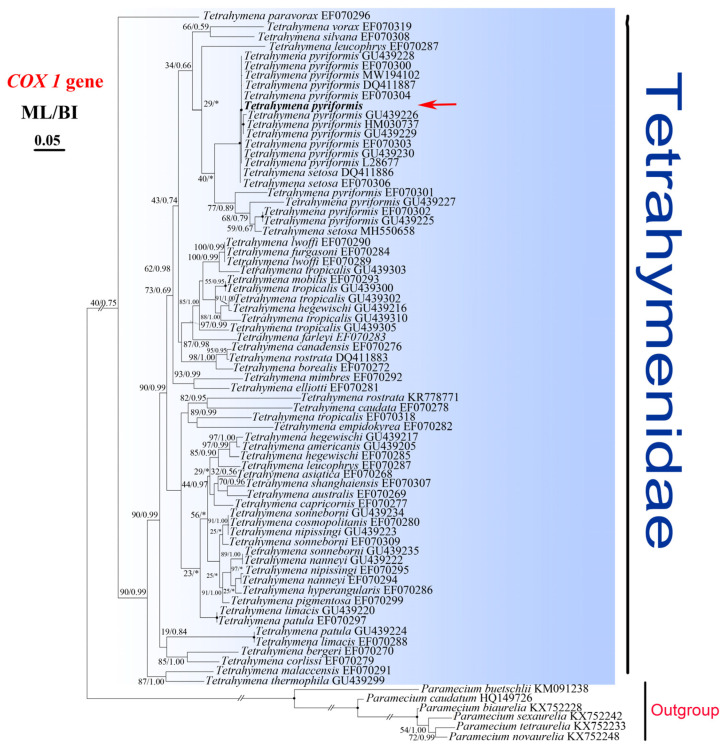
Maximum likelihood (ML) tree inferred from the *cox1* gene sequences showing the systematic position of *Tetrahymena pyriformis* (in bold and red arrow). ‘*’ indicates topologies that differ between the ML and BI phylogenies. The scale bar corresponds to five substitutions per 100 nucleotide positions.

The topologies of BI trees and ML trees were similar, so only the ML trees were shown in Figure 5, with branch support values for both analyses. As shown in the consensus topology, three groups (‘borealis group’, ‘australis group’, ‘riboset group’, and ‘paravorax group’) were recognizable within the non-monophyletic group of Tetrahymenidae. The Harbin population of *T. pyriformis* clustered in the ‘borealis group’, and clusters in the clade containing other populations of *T*. *pyriformis*. The newly sequenced *cox1* gene of the Harbin population differed from the sequences of the previously isolated strains of *T. pyriformis* by one to three nucleotides.

### 3.4. Experimental Infection

To replicate the aquarium’s environmental conditions at varied temperatures and pH levels, we used *T. pyriformis* to infect abraded guppies (Figure 1).

**Figure 6:**
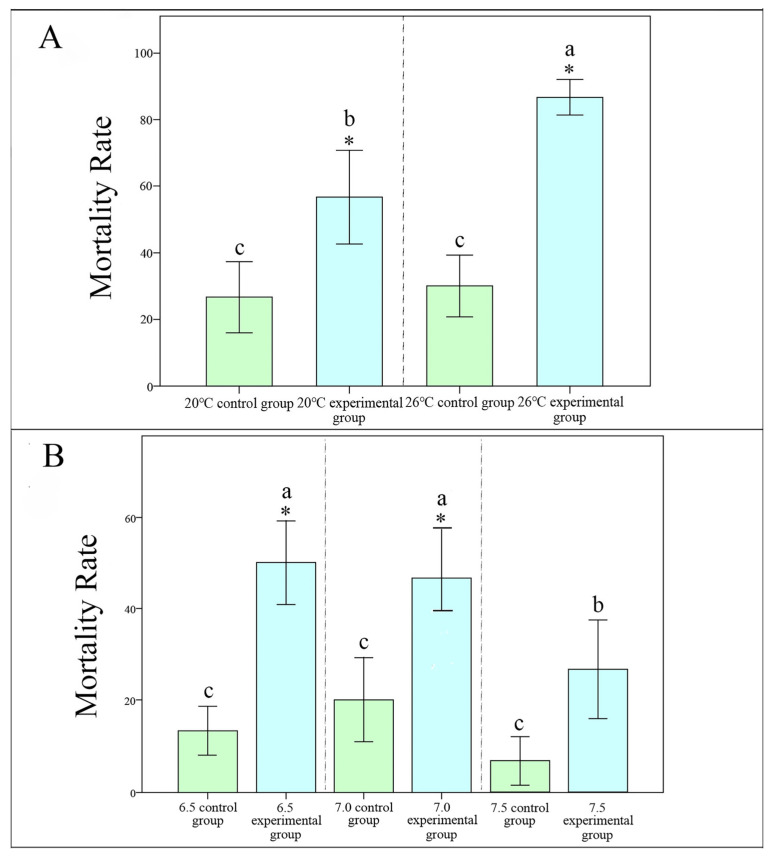
(**A**): Mortality Rate of guppy (*t*-test and Duncan’s multiple range test, *p* < 0.05). ‘*’ indicates that there is a significant difference between the experimental (a and b) and control (c) groups. (**A**) Temperature treatment group. (**B**) pH treatment group.

A one-way ANOVA conducted on the temperature-treated groups revealed a *p*-value of 0.01, indicating a highly statistically significant result. Following this, we performed a Duncan’s multiple range test to further assess the differences among groups. As shown in the fish mortality charts (Figure 6A), in the control group at 20 °C, the mortality rate was 30%, while in the 20 °C experimental group, the mortality rate was 60%. After conducting a Duncan’s multiple range test on the mortality rate of fish, we obtained a *p*-value of 0.011, which indicated a statistically significant result. In the control group at 26 °C, the mortality rate was 35%, while in the 26 °C experimental group, the mortality rate was 85%. After conducting a Duncan’s multiple range test on the mortality rate of fish, we obtained a *p*-value of 0.001, which indicated a statistically significant result. In the experimental group at 20 °C, the mortality rate was 60%, while in the 26 °C experimental group, the mortality rate was 85%. After conducting a Duncan’s multiple range test on the mortality rate of fish, we obtained a *p*-value of 0.011, which indicated a statistically significant result.

A one-way ANOVA conducted on the pH-treated groups revealed a *p*-value of 0.03, indicating a statistically significant result. Following this, we performed a Duncan’s multiple range test to further assess the differences among groups. As shown in the fish mortality charts (Figure 6B), in the control group of 6.5, the mortality rate was 15%, while in the experimental group of 6.5, the mortality rate was 50%. After conducting a Duncan’s multiple range test on the mortality rate of fish, we obtained a *p*-value of 0.02, which indicated a statistically significant result. In the control group of 7.0, the mortality rate was 20%, while in the experimental group of 7.0, the mortality rate was 50%. After conducting a Duncan’s multiple range test on the mortality rate of fish, we obtained a *p*-value of 0.017, which indicated a statistically significant result. In the experimental group of 6.5, the mortality rate was 50%, while in the 7.5 experimental group, the mortality rate was 30%. After conducting a Duncan’s multiple range test on the mortality rate of fish, we obtained a *p*-value of 0.032, which indicated a statistically significant result.

### 3.5. Diversity and Composition of Microflora

A total of 30 samples were sequenced, including triplicate repetitions of control and experimental groups under treatments at temperatures of 20 °C and 26 °C and pH levels of 6.5, 7.0, and 7.5. The bacterial samples were derived from the simulated tanks inhabited by guppies. After noise reduction and de-chimerism, a total of 3480 OTUs and 69,624 sequences were obtained from the generated OTU table.

A one-way ANOVA conducted on the number of OTU species in temperature groups revealed a *p*-value of 0.015, indicating a statistically significant result. Following this, we performed a Duncan’s multiple range test to further assess the differences among groups. In the control group at 20 °C, the numbers of OTUs were 249, while in the 20 °C experimental group, the numbers of OTUs were 323, and after conducting a Duncan’s multiple range test, we obtained a *p*-value of 0.029, which indicated a statistically significant result. In the control group at 26 °C, the numbers of OTUs were 257, while in the 26 °C experimental group, the numbers of OTUs were 283, and after conducting a Duncan’s multiple range test, we obtained a *p*-value of 0.013, which indicated a statistically significant result.

A one-way ANOVA conducted on the number of OTU species in pH groups revealed a *p*-value of 0.045, indicating a statistically significant result. Following this, we performed a Duncan’s multiple range test to further assess the differences among groups. In the control group of 6.5, the numbers of OTUs were 292, while in the 6.5 experimental group, the numbers of OTUs were 304, and after conducting a Duncan’s multiple range test, we obtained a *p*-value of 0.042, which indicated a statistically significant result. In the control group of 7.0, the numbers of OTUs were 251, while in the 7.0 experimental group, the numbers of OTUs were 319, and after conducting a Duncan’s multiple range test, we obtained a *p*-value of 0.050, which indicated a statistically significant result. In the control group of 7.5, the numbers of OTUs were 242, while in the 7.5 experimental group, the numbers of OTUs were 287, and after conducting a Duncan’s multiple range test, we obtained a *p*-value of 0.067, which did not indicate a statistically significant result.

Notably, at various temperatures, 1555 OTUs and 27,709 sequences were identified across 10 phyla and 14 classes. Among these, Proteobacteria accounted for the highest proportion of OTU species at the phylum level, totaling 56%, followed by Bacteroidetes at 38%. Regarding classes, the most prevalent OTU species were Gammaproteobacteria and Bacteroidia, collectively representing 38%. Similarly, at different pH values, 1925 OTUs and 41,915 sequences were observed, spanning 10 phyla and 16 classes. Notably, Proteobacteria constituted the largest proportion of OTU varieties at the phylum level, mirroring the temperature treatment group at 60%, followed by Bacteroidetes at 28%. Notably, at the class level, the highest number of OTU species was Gammaproteobacteria, accounting for 39%.

**Figure 7:**
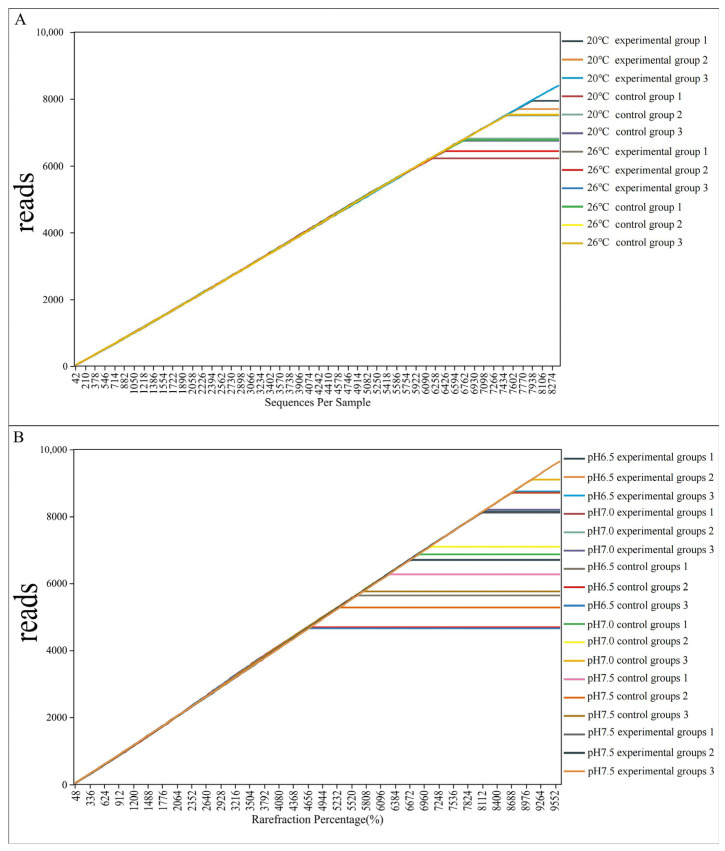
Dilution curve. (**A**) Temperature treatment group. (**B**) pH treatment group.

Based on the results of various variables, we have generated separate dilution curves (Figure 7). It is evident that as the number of reads increases, the species diversity curve initially rises sharply but eventually levels off. This indicates that, despite a continued increase in the number of sequenced samples, the species count has reached a stable point. Consequently, the sample size for this experiment appears to be both adequate and justified.

The taxonomic sequence information of phylum, class, order, family, genus, species, and OTU in the OTU table was counted, and the relative abundance of each taxon was calculated. At the same time, the taxa with relative abundance above 1% (default value) were selected, and the 15 OTUs with the highest abundance were selected to draw the relative abundance microflora stack map.

**Figure 8:**
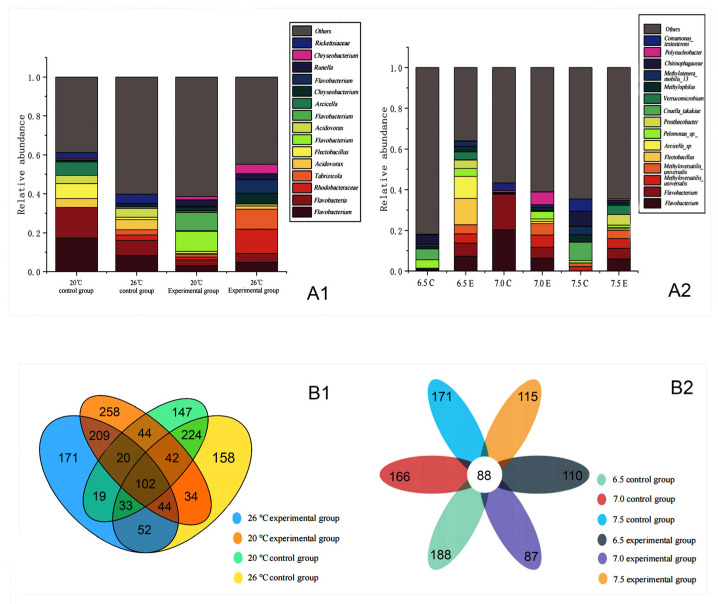
(**A**): The colony stacking map of the top 15 OTUs. (**A1**) Temperature treatment group. (**A2**) pH treatment group. In the A2, ‘C’ stands for the control group, ‘E’ for the experimental group. (**B**): Common and unique OTU Venn diagrams. (**B1**) Temperature treatment group. (**B2**) pH treatment group.

Under different temperature treatments (Figure 8(A1)), at 20 °C and 26 °C control groups, the OTUs with high relative abundance were *Flavobacteria bacterium BAL38*, respectively. Moreover, the abundance of this bacteria was higher in the 20 °C control group compared to the 26 °C control group. Compared with the experimental group, the unique OTU in the control group belongs to the Rickettsiaceae flora. In the 20 °C experimental group, the relative abundance of *Flavobacterium aquatile LMG 4008* was higher than its abundance in the 20 °C control group, the 26 °C control group, and the 26 °C experimental group. In the 26 °C experimental group, a high percentage of OTUs belonged to Rhodobacteraceae, *Tabrizicola*, *Flavobacterium*, and *Chryseobacterium*, respectively.

Under different pH treatments (Figure 8(A2)), in the experimental groups at pH 6.5, 7.0, and 7.5, the abundance of *Flavobacterium* and *Methyloversatilis universalis* was higher compared to the control groups. In the pH 6.5 experimental group, the flora with the highest abundance of OTUs were *Flectobacillus* and *Arcicella* sp. *7A-626*.

The Venn diagram of the temperature treatment group showed that there were 224 species of microflora in the control group at 20 °C and 26 °C and 209 species in the experimental group (Figure 8(B1)). At the same temperature, there were 44 species of bacteria in the control group and the experimental group at 20 °C and 52 species at 26 °C. The results showed that the addition of *T. pyriformis* would have a great influence on the species of microflora. The Venn diagram of the pH treatment group showed that there were 88 species of flora in each group, and the number of endemic florae in the pH 6.5 control group was the most, which was consistent with the α diversity map (Figure 8(B2)). Compared with the control group, the diversity of microflora in the experimental group with *T. pyriformis* was smaller.

**Figure 9:**
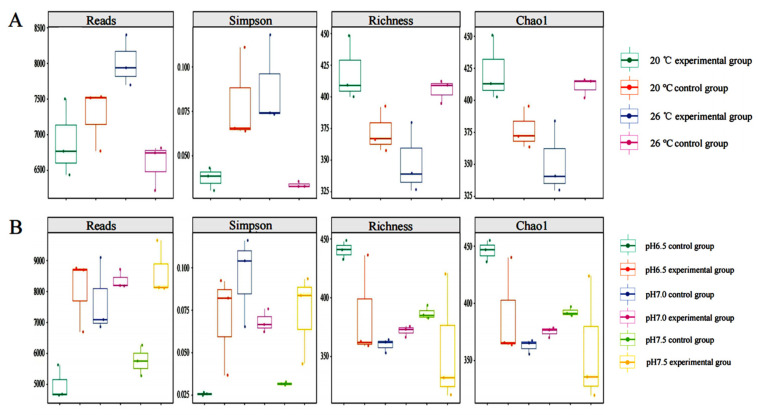
Alpha diversity box diagram. (*p* < 0.05) (**A**) Temperature treatment group. (**B**) pH treatment group.

Overall, the alpha diversity index indicates noticeable differences in bacterial communities across various treatment groups (Figure 9). Specifically, within different temperature treatment groups, the α diversity index was higher at 26 °C than that at 20 °C in the control group; yet, in the experimental group with *T. pyriformis*, the microflora diversity at 20 °C surpassed that at 26 °C. Likewise, in different pH treatment groups, the diversity of the *T. pyriformis* experimental group significantly diverged from that of the control group. These outcomes indicate the dynamic interaction between microflora and *T. pyriformis* at distinct temperatures and pH levels, where *T. pyriformis*, either fosters or curbs the growth of certain bacteria.

In this study, the relative abundance of bacteria (OTU clustering) was related to the abundance of *T. pyriformis* in water and the mortality of fish, as well as different physical factor variables (temperature and pH). The correlation heat map was created to show the correlation between more than 20 species of bacteria and fish mortality, the abundance of *T. pyriformis*, and different physical factors.

**Figure 10:**
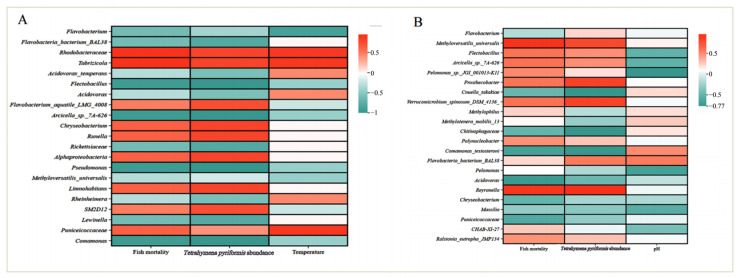
Heat map of correlation between OTU flora with higher abundance and fish mortality, abundance of *Tetrahymena pyriformis*, and environmental factors. (**A**) Temperature treatment group. (**B**) pH treatment group.

In different temperature treatment groups (Figure 10A), correlation heat maps showed that Rhodobacteraceae, *Tabrizicola*, and Puniceicoccaceae were positively correlated with fish mortality, *T. pyriformis* abundance, and temperature. *Tabrizicola* is assigned to the Rhodobacteraceae, and it is also a flora of OTUs with high abundance. The microflora of the three OTUs has a strong positive correlation with temperature. *Chryseobacterium*, *Runella*, Alphaproteobacteria, *Limnohabitans*, and Puniceicoccaceae were positively correlated with fish mortality and *T. pyriformis* abundance. *Flectobacillus*, *Arcicella* sp.* 7A-626*, *Pseudomonas*, *Comamonas*, *Flavobacterium*, and *Methyloversatilis universalis* were negatively correlated with fish mortality, *T. pyriformis* abundance, and temperature. There was a strong negative correlation between *Flectobacillus*, *Arcicella* sp.* 7A-626*, *Pseudomonas*, *Comamonas*, and *T. pyriformis* abundance.

In different pH treatment groups (Figure 10B), the correlation heat maps showed that *Methyloversatilis universalis*, *Flectobacillus*, *Arcicella* sp. *7A-626*, *Prosthecobacter*, *Reyranella*, and *Flavobacteria bacterium BAL38* were positively correlated with fish mortality and *T. pyriformis* abundance. *Cnuella takakiae*, *Chitinophagaceae*, *Comamonas testosteroni*, *Acidovorax*, *Chryseobacterium*, *Massilia*, and Puniceicoccaceae were negatively correlated with *T. pyriformis* abundance. *Methyloversatilis universalis*, *Flectobacillus*, *Arcicella* sp. *7A-626*, *Prosthecobacter*, *Reyranella*, and *Flavobacteria acterium BAL38* were positively correlated with fish mortality and *T. pyriformis* abundance. *Reyranella*, like *Tabrizicola* in the temperature treatment group, is also a member of the Rhodospirillaceae family, and it is positively correlated with *T. pyriformis* abundance. *Flavobacteria bacterium BAL38* belongs to Flavobacterium, and most of them are pathogenic, which may be the reason why it is positively correlated with fish mortality and *T. pyriformis*.

*Cnuella takakiae*, *Chitinophagaceae*, *Comamonas testosterone*, *Acidovorax*, *Chryseobacterium*, *Massilia*, and Puniceicoccaceae were negatively correlated with *T. pyriformis* abundance. In the temperature treatment group, *Chryseobacterium* was positively correlated with *T. pyriformis* abundance, and it also had pathogenicity, but in the pH treatment group, it was negatively correlated with *T. pyriformis* abundance, which may be due to the influence of pH. In this study, there is a negative correlation between *Chryseobacterium* and pH.

## 4. Discussion

The morphological characteristics and stages in the full polymorphic life history, including the microstome and macrostome, of the present population of *T. pyriformis* are similar to those described before [41,42,43], except that the Harbin population has a smaller body size and habitat (facultative parasitic vs. non-parasitic form), compared with previous populations of *T. pyriformis*. In the consensus topology (Figure 4), three groups (‘borealis group’, ‘australis group’, and ‘riboset group’) are recognizable within the monophyletic group of Tetrahymenidae, which is consistent with previous studies [44,45,46,47]. The Harbin population of *T. pyriformis* groups with other populations of *T. pyriformis*, which supports the morphological identification of the Harbin population of this species.

The interaction between protozoa and bacteria is thought to play an important role in the food web of the aquatic environment. Ciliates may change their reproductive and feeding behavior according to the abundance and diversity of bacterial microbiota in the environment [48,49]. In order to study the relationships between mortality of fish, species, and abundance of *T. pyriformis* and bacteria under the influence of different environmental factors, we simulated the aquatic environment of the aquarium, set different temperature and pH variable treatment groups for infected guppies with *T. pyriformis*, and combined the methods of eDNA metabarcoding and qPCR. And this method can also be used to monitor the abundance of parasites in aquariums for follow-up protective measures.

The method of environmental DNA metabarcoding has become more popular in recent years, and many scholars use this method for environmental monitoring to prove the feasibility of this method [18,50]. Monitoring based on environmental DNA is not only fast and convenient but also a non-destructive sampling method. Traditional methods of monitoring parasites, such as anaesthesia and bath therapy for aquaculture, are destructive to the aquarium environment and ornamental fish. The use of an eDNA-based monitoring method can not only reduce the sampling cost but also reduce the mortality of ornamental fish treated for monitoring. This study set up the infection situation of *T. pyriformis* and found that higher temperature and lower pH were disadvantageous to ornamental fish culture, and these conditions were more likely to promote disease outbreak and death of guppies.

In the variable representing temperature in this study, two treatment groups were set at 20 °C and 26 °C. Guppy, being a tropical fish, thrives in warmer environments. In the 26 °C group, which represented the warmer and more favorable conditions, guppies experienced a higher mortality rate compared to those at 20 °C. This phenomenon is believed to be due to the increased susceptibility of guppies to bacterial or parasitic infections as a result of the higher temperature and potential physical abrasions. The diseased fish treated at 26 °C also had more infections (excessive mucus on the body surface, erythema, etc.). The pH sets three variable processing groups of 6.5, 7.0, and 7.5, which are the minimum, intermediate, and maximum values of pH suitable for guppies, respectively. In the pH treatment group, the lower the pH, the higher the mortality of guppies, which is consistent with guppies’ habit of living in weakly alkaline water.

It was found that a large amount of active *T. pyriformis* was found on the body surface (body surface mucus, fish gills, fin tube, exposed wound) of infected guppies, but no large amount of *T. pyriformis* was observed in the visceral part. *Tetrahymena pyriformis* obtained from diseased fish was observed in vivo, and it was found that there were pigment particles of fish meat in the food vesicles. Tissue sections showed that pathological changes occurred in all tissues of diseased fish, but the number of *T. pyriformis* was getting lower and lower from outside to inside. The above results showed that the exposed sites, such as the skin, were the breakthrough of *T. pyriformis* infection, thus reaching the internal organs through the circulatory system, which is consistent with the results of previous studies [20,51]. During the experiment, it was observed that smaller fish exhibited earlier mortality compared to larger fish. Additionally, a positive correlation was noted between the maturity of guppies and their resistance levels, indicating that as guppies matured, their resistance increased.

Ciliate infections and microbial species mutually reinforce each other, both influenced by environmental and cultural conditions. Research indicates that infectious diseases may result from multiple pathogens rather than a singular pathogen [20]. Disease outbreaks in aquaculture can be caused by a variety of pathogens, including viruses, bacteria, and fungi, as well as external and internal parasites [52]. Infection and environmental factors were considered to be closely related, and co-infection may be one of the most common forms of connection between ciliates, protozoa, parasites, and bacteria in the aquatic environment [53]. In recent years, research on the relationship between ciliates and bacterial microflora in aquaculture systems has gradually increased. Understanding and guiding the processes of these interactions can help prevent disease outbreaks in aquatic systems. By understanding the nature of this interaction, aquarium owners can change strategies for dealing with *Tetrahymena* disease, such as changing aquaculture water, appropriately cooling and increasing pH values, adding probiotics, etc., using these methods to reduce the abundance of *Tetrahymena* without the need for synthetic drugs or chemotherapy.

In the association of bacterial communities in the temperature treatment group, Rhodobacteraceae, *Tabrizicola*, and Puniceicoccaceae were positively correlated with fish mortality, *T. pyriformis* abundance, and temperature. *Tabrizicola* belongs to the Rhodobacteraceae, and they also accounted for a high proportion of the microflora in the treatment group. Rhodobacteraceae is an aquatic bacterium that often thrives in the marine environment [54]. *Tabrizicola* is a pure chemonutrient genus of gram-negative, aerobic, non-motor, and rod-shaped bacteria [55]. *Chryseobacterium*, *Runella*, Alphaproteobacteria, *Limnohabitans*, and Puniceicoccaceae were positively correlated with fish mortality and *T. pyriformis* abundance. Most members of *Chryseobacterium* occur in aquatic environments or food, and many of these strains are pathogenic to humans and animals [56,57]. This group of bacteria was found in fish, and its members should be regarded as potential emerging pathogens in various fish and frog species, culture conditions, and geographical areas [58]. Therefore, under different temperature treatments in this study, a strong correlation was observed among significant quantities of *Chryseobacterium*, high fish mortality rates, and elevated *T. pyriformis* abundance.

*Flectobacillus*, *Arcicella* sp. *7A-626*, *Pseudomonas*, *Comamonas*, *Flavobacterium*, and *Methyloversatilis universalis* were negatively correlated with fish mortality, *T. pyriformis* abundance, and temperature. *Pseudomonas* members were abundant in all major natural environments (terrestrial freshwater and marine) and had a significant ability to degrade a variety of substrates, including aromatic compounds, halogenated derivatives, and stubborn organic residues [59]. Some bacteria in the family, such as *Pseudomonas aeruginosa*, could infect a range of organisms, including plants, nematodes, fruit flies, zebrafish, and various mammals [60,61,62,63,64,65]. Most members of *Flavobacterium* are pathogenic to freshwater fish, and some were occasionally isolated from diseased freshwater fish [66]. However, in this study, it was negatively correlated with fish mortality, which might be due to the interaction between *T. pyriformis* and these bacteria. All these bacteria were negatively correlated with *T. pyriformis*, which may be due to the phagocytosis of *T. pyriformis*. It is also possible that temperature and *T. pyriformis* affect them together. Bacteria may be inactivated and consumed by ciliates in the environment [67]. There is evidence that *E. coli* Migula, 1895, is pathogenic to humans but not to fish. They have been found in the intestines, muscles, skin, and gills of fish [68] and can be significantly reduced by ciliates such as *T. pyriformis* [69].

In different treatment groups, *Comamonas* species were all negatively correlated with the abundance of *T. pyriformis*. *Comamonas* species are a group of gram-negative, non-fermenting, and rod-shaped bacteria. It has been found that *Comamonas* strains may have specific genomic characteristics at the genus level and play a certain ecological role in different habitats. The virulence factors in *Comamonas* have high species specificity [70]. Members of *C. testosteroni* are environmental microorganisms that usually exist in contaminated environmental samples [71]. It has been found that it has a potential driving mechanism for the degradation of polycyclic aromatic hydrocarbons (PAHs) [72,73]. The above results showed that both *C. testosteroni* and *Comamonas* had great effects on the aquatic environment. In this study, *Comamonas* was related to the low level of *T. pyriformis* abundance and might be related to these characteristics. There was a negative correlation between *Comamonas* and temperature. The present study showed a positive correlation between *Comamonas testosteroni* and pH.

## 5. Conclusions

We replicated an indoor ornamental fish farming environment and used eDNA extraction and qPCR technology to study tetrahymenosis under varying temperature and pH conditions, aiming to investigate the correlation between mortality guppies, *T. pyriformis*, and related bacterial microflora across these different settings. Our investigation revealed positive correlations between *Tabrizicola*, Puniceicoccaceae, *Chryseobacterium*, fish mortality, and the abundance of *Tetrahymena pyriformis*. Conversely, *Comamonas* displayed a negative correlation with fish mortality in both groups. Notably, certain bacteria exhibited an inverse correlation with fish mortality and the abundance of *T. pyriformis* under diverse physicochemical conditions, demonstrating that the relationship between bacteria and *T. pyriformis* could be influenced by environmental factors. In addition, the morphological identification and phylogenetic analysis of *T. pyriformis* were also carried out. The pathological features were investigated using hematoxylin-eosin staining.

## Data Availability

*Cox1* gene sequences were downloaded from Genbank at https://www.ncbi.nlm.nih.gov/ (accessed on 20 July 2024).
